# Anti-NMDA-receptor antibody detected in encephalitis, schizophrenia, and narcolepsy with psychotic features

**DOI:** 10.1186/1471-244X-12-37

**Published:** 2012-05-08

**Authors:** Ko Tsutsui, Takashi Kanbayashi, Keiko Tanaka, Shuken Boku, Wakako Ito, Jun Tokunaga, Akane Mori, Yasuo Hishikawa, Tetsuo Shimizu, Seiji Nishino

**Affiliations:** 1 Akita University, Department of Neuropsychiatry, Akita, Japan; 2 Kanazawa Medical University, Department of Neurology, Ishikawa, Japan; 3 Hokkaido University, Department of Neuropsychiatry, Sapporo, Japan; 4 University of South Carolina, Department of Exercise Science, Columbia, SC, USA; 5 Akita Kaiseikai Hospital, Department of Psychiatry, Akita, Japan; 6 Stanford University, Sleep and Circadian Neurobiology Laboratory, Palo Alto, CA, USA

## Abstract

**Background:**

Causative role of encephalitis in major psychotic features, dyskinesias (particularly orofacial), seizures, and autonomic and respiratory changes has been recently emphasized. These symptoms often occur in young females with ovarian teratomas and are frequently associated with serum and CSF autoantibodies to the NMDA receptor (NMDAR).

**Methods:**

The study included a total of 61 patients from age 15 to 61 and was carried out between January 1, 2005, and Dec 31, 2010. The patients were divided into the following three clinical groups for comparison. Group A; Patients with typical clinical characteristics of anti-NMDAR encephalitis. Group B; Patients with narcolepsy with severe psychosis. Group C; Patients with schizophrenia or schizo-affective disorders.

**Results:**

Ten out of 61 cases were anti-NMDAR antibody positive in typical encephalitis cases (group A: 3 of 5 cases) and cases in a broader range of psychiatric disorders including narcolepsy (group B: 3 of 5 cases) and schizophrenia (group C: 4 of 51 cases).

**Conclusion:**

In addition to 3 typical cases, we found 7 cases with anti-NMDAR antibody associated with various psychotic and sleep symptoms, which lack any noticeable clinical signs of encephalitis (seizures and autonomic symptoms) throughout the course of the disease episodes; this result suggest that further discussion on the nosology and pathophysiology of autoimmune-mediated atypical psychosis and sleep disorders is required.

## Background

Recently, causative role of encephalitis in major psychotic features, dyskinesias (particularly orofacial), seizures, and autonomic and respiratory changes has been emphasized [1,2]. These symptoms often occur in young females with ovarian teratomas, who have good responses to tumor surgery and immunotherapy [3-6]. Anti-NMDA-receptor (NMDAR) encephalitis is suggested in many of these cases as they are frequently associated with serum and CSF autoantibodies to the NMDA receptor (NMDAR) [6].

A stereotypical clinical course during phases is noted for the patients with Anti-NMDAR encephalitis [7]; a non-specific flu-like prodrome (subfebrile temperature, headache, fatigue) is always followed by a psychotic stage with bizarre behavior, disorientation, confusion, paranoid thoughts, visual or auditory hallucinations and memory deficits. Acute onsets of atypical psychosis are usually considered initially, and the patients are often admitted to psychiatric centers. Organic brain disease is considered only after the patients develop seizures, autonomic instability, dyskinesias, or decreased level of consciousness [6,8,9].

In the current study, we indentified 3 typical Japanese anti-NMDAR encephalitis cases. In addition, we found 7 Japanese cases with anti-NMDAR antibody with various psychotic and sleep symptoms, who lack any noticeable clinical signs of encephalitis (seizures and autonomic symptoms) throughout the courses of the disease episodes. These patients exhibited two distinct clinical characteristics, and we report clinical symptoms of these cases along with the typical cases.

## Method

The study included a total of 61 patients aged 15 to 61 years. They were studied in the Department of Neuropsychiatry, Akita University Hospital and related hospitals between January 1, 2005, and Dec 31, 2010. The patients were divided into 3 clinical groups for comparison.

Group A had typical clinical characteristics of anti-NMDAR encephalitis, beginning with psychiatric symptoms, followed by subsequently occurring seizures and disturbances of consciousness (Table 1). In order to examine the specificity of the anti-NMDAR antibody involvement in these cases, we also examined the prevalence of antibody positivity in other neurologic and psychotic patients without signs of encephalitis. Five narcolepsy with severe psychosis cases were examined and also included (group B), because autoantibody-mediated mechanisms (anti-Ma2, anti-aquaporine 4 antibodies) are suspected in some secondary narcolepsy cases [10,11]. In addition, several research groups recently reported that a swine flu (H1N1) vaccination increased the incidence of hypocretin-deficient narcolepsy [12]. The antibody levels of 10 narcolepsy cases without psychosis were additionally measured for comparison with group B. We also examined the antibody in 51 patients with schizophrenia or schizo-affective disorders (group C). Group C was subdivided into (c-1) schizophrenia accompanied with convulsion [13], (c-2) atypical symptoms of psychosis, and (c-3) resistance to pharmacological treatments with relatively good responses to modified electric convulsion treatment (mECT).

**Table 1:** Characteristics and clinical features of 10 NMDAR antibody positive patients and negative controls

**Group**	**Age, Sex**	**Diagnosis**	**Psychotic symptoms**	**Epileptic attack**	**EEG, sleep symptoms**	**Treatments**	**Others**	**Labolatory for NMDAR antibodies measurements**	**References**
1(A) encephalitis group	18/F	anti-NMDAR encephalitis	talkativeness, hyperactivity, bizzare behavoir	generalized tonic-clonic seizure	normal	steroid pulse therapy	slight high density inside the bilateral temporal regions (FLAIR, T2)	Dr Dalmau (University of Pennsylvania)	[14]
2(A) encephalitis group	24/M	anti-NMDAR encephalitis	delusion, catalepsy, palilalia	generalized tonic-clonic seizure	11Hz, alpha wave, (after stroid pulse therapy)	steroid pulse therapy		Dr Dalmau (University of Pennsylvania)	[15]
3(A) encephalitis group	27/F	anti-NMDAR encephalitis	substupor, catatonia	unspecified seizure	normal	antipyschotics, m-ECT	after m-ECT, ataxia, nystagmus and agraphia were pointed out	Dr Tanaka(Kanazawa Medical University)	
N(A) type antibody-negative (n=2)	59/F, 25/M	Limbic encephalitis suspected	stupor, catatonia, hyperactivity, bizzare behavoir,reference delusion	generalized tonic-clonic seizure (n=1)	spike and wave complex (n=1), diffuse slow alpha wave (n=2)	antipyschotics, m-ECT (n=1)	prodromal ful-like symptoms (n=1)		
4(B) narcolepsy with psychosis group	61/M	Narcolepsy, Parkinson's disease, Delusional disorder(297.1)(F22.0)	visual hallucination, persecutory delusion, delusion of jealousy	(-)	short sleep latency, sleep onset REM periods	antipyschotics, m-ECT	resting tremor, hypocretin deficient narcolepsy	Dr Tanaka(Kanazawa Medical University)	[16,17]
5(B) narcolepsy with psychosis group	37/F	Narcolepsy, Schizophrenia(Paranoid type 295.30)(F20.0)	visual and auditory hallucinations, delusion	(-)	short sleep latency, sleep onset REM periods	antipyschotics	hypocretin deficient narcolepsy	Dr Tanaka(Kanazawa Medical University)	
6(B) narcolepsy with psychosis group	24/F	Narcolepsy, Schizophrenia(Paranoid type 295.30)(F20.0)	auditory hallucination, delusion, agitation, aggression,	(-)	short sleep latency, sleep onset REM periods	antipyschotics	hypocretin deficient narcolepsy	Dr Tanaka(Kanazawa Medical University)	
N(B) type antibody-negative (n=2)	25/F, 35/M	Narcolepsy and psychosis (25/F)(298.9)(F29), Narcolepsy and Schizophrenia (35/M)(F20.1)(295.10)	agitation, aggression, auditory hallucination	(-)	short sleep latency, sleep onset REM periods	antipyschotics	hypocretin deficient narcolepsy		
7(C) psychiatric sympotoms	53/F	Schizophrenia(Catatonic type:295.20)(F20.24), Mental Reterdation(F71.0)	premenstrual tension, atypical pyschosis	generalized tonic-clonic seizure	normal	antipyschotics, m-ECT		Dr Tanaka(Kanazawa Medical University)	
8(C) psychiatric sympotoms	34/F	Schizoaffective disorder(295.70)(bipolar type:F25.0)	delusion, auditory hallucination, talkativeness, hyper activity, aggression	complex partial seizure	normal	valproic acid	reccurent ovarian cyst, operations were performed	Dr Tanaka(Kanazawa Medical University)	
9(C) psychiatric sympotoms	30/F	Schizophrenia(Disorganized type:295.10)(F20.12)	acoustic hyperesthesia, irritation	(-)	normal	antipyschotics, m-ECT	oral dyskinesia	Dr Tanaka(Kanazawa Medical University)	[18]
10(C) psychiatric sympotoms	26/F	Schizophrenia(Catatonic type:295.20)(F20.25)	hallucination, delusion, depressive mood, hyper activity	(-)	normal	antipyschotics, m-ECT	ovarian tumor, operation was performed	Dr Dalmau (University of Pennsylvania)	[19]
N(C) type antibody-negative (n=47)	15-72y, M:F=10:37	Schizophrenia(295.30,295.20)(F20.0.F20.2), Schizoaffective disorder(295.70), brief psychotic disorder(298.8)(F23.1)	hallucination, delusion, depressive mood, hyper activity, bizzare behavoir, catatonia	generalized tonic-clonic seizure (n=10)	abnormal EEG (n=6)	antipyschotics, m-ECT	ovarian tumor (n=6), convulsion&ovarian tumor (n=2)		

Antibody detection was performed by Dr. Dalmau's laboratory in cases 1, 2, 10 and by Dr. Tanaka’s laboratory for the others in Table 1. Case reports for 1, 2, 4, 9, 10 were previously published [14-19].During the initial study with Dr. Dalmau, we came across several patients with positive antibody but without any symptoms of encephalitis (in group C). We therefore extended the study and measured the anti-NMDAR antibody in additional cases by ourselves with a comparative method [20,21]. The plasma and CSF were tested blind to diagnostic status.

The study was approved by the Akita University ethics committee and all patients gave informed written consent prior to the study.

## Results

Each antibody positive case is described, while the negative cases are summarized in Table 1. Psychiatric disorders, behavioral disorders, movement disorders, or sleep disorders are mainly presented. The DSM-IV diagnostic codes are included. The details of the clinical characteristics of the representative 3 cases of each group are presented in the text. Ten cases were anti-NMDAR antibody positive; 3 of 5 cases of typical encephalitis (group A), 3 of 5 cases with a broader range of psychiatric disorders including narcolepsy (group B) and 4 of 51 cases with schizophrenia or schizo-affective disorders (group C).

### (Group A) Typical clinical pictures of anti-NMDAR encephalitis

We reviewed a case of acute limbic encephalitis (NMDAR antibody was detected retrospectively) diagnosed after improvement of psychotic symptoms by mECT. This case was first diagnosed as schizophrenia based on catatonia-like symptoms, auditory hallucinations, and delusions. Two other positive cases presented with psychosis, convulsions and were treated with steroid pulse therapy.

#### Case report

The patient (case 3) was a 27-year-old female and had no previous psychiatric, neurological, or family history of the disease. After a common cold, the patient had hypobulia, insomnia, and seizure-like episodes. In addition, the patient exhibited strange and incoherent behaviors. During her first visit to our department, she was substuporous. After hospitalization, the existence of auditory hallucination and persecutory delusions were also strongly suggested. Blood examinations, brain MRI, and EEG showed no abnormality. Therefore at first we diagnosed the patient as having acute schizophrenia. Since her psychotic symptoms were refractory to antipsychotic medication and adverse effects were severe, we applied mECT. The psychotic symptoms improved remarkably by this treatment. Accessibility was also improved, and we were able to identify various neurological and psychiatric symptoms including aphasia, agraphia, constructional apraxia, retrograde amnesia, personality change, and disinhibition. Laboratory examinations involving CSF and brain SPECT were performed. Brain SPECT showed decreased blood flux in her left limbic system and in the inside of the left temporal lobe. From the symptoms, clinical course, rareness of abnormality in various examinations, and the findings of brain SPECT, we diagnosed her as having limbic encephalitis. Thereafter her symptoms naturally improved without antipsychotics, and her mental condition has been kept stable and healthy.

Because her clinical picture resembled that of the recently reported anti-NMDAR encephalitis, we examined the possible association. An archived plasma sample from her initial presentation was submitted to Kanazawa Medical University, where the antibody against NMDAR was detected in the sample; the final diagnosis was anti-NMDAR encephalitis. Examinations for tumors were not performed at initial hospitalization.

The discrimination between functional (endogenous) psychosis and NMDAR encephalitis is sometimes very difficult. Therefore, we have to consider the possibility of anti-NMDAR encephalitis, especially when relatively young women are suffering acutely from psychotic symptoms.

We provided immunotherapy in case 1 and 2 but not in case 3 because non-herpes limbic encephalitis was initially suspected for the former two cases.

Another two subjects presented typical clinical pictures of anti-NMDAR encephalitis, beginning with psychotic symptoms, followed by seizures and subsequent disturbances of consciousness. However, these two subjects with similar clinical pictures to case 3 were negative for the anti-NMDAR antibody (Table 1, group NA).

### (Group B) Narcolepsy cases with severe psychosis

We had previously reported a case with Parkinson’s disease (PD) comorbid with hypocretin (orexin) deficient narcolepsy [16,17]. In this patient, severe psychosis presented subsequently to the diseases above, and has been treated by mECT in addition to anti-psychotics with a successful outcome. NMDAR antibody was detected retrospectively. The two other positive cases had narcolepsy with severe psychosis without neurodegenerative disease.

#### Case report

A 58-year-old male (case 4) with mild PD for 15 years was admitted to a hospital due to a sleep attack in 2004. He suffered from excessive daytime sleepiness (EDS) at high school, however, the patient had not been diagnosed or treated at that time. The patient had never had cataplexy (sudden loss of muscle tonus due to emotional trigger). At age 43, the patient had tremors in his left fingers and at age 45 was diagnosed with PD. The patient was hit by a motor vehicle due to EDS at age 55 and started experiencing frequent hypnagogic hallucinations with abnormal limb movements. The patient had Hoehn-Yahr stage 2 Parkinsonism and scored 14 in the unified PD rating scale part III. His epworth sleepiness scale result was 19/24 (normal range < 11/24). In the multiple sleep latency test (MSLT), mean sleep latency was shortened to 2 min (normal range >8 min) and sleep onset REM periods were present in all four naps. HLA was positive for DR15 (2) as typical for idiopathic narcolepsy. CSF hypocretin (orexin) concentration was very low (86 pg/ml, normal range >200 pg/ml). The patient was treated with methylphenidate (MPH) for EDS in addition to medications for PD. During the next two years, his condition was good. Thereafter, the patient became delusional and suffered from auditory hallucinations. MPH and medications for PD were stopped, and anti-psychotics were used. However, the serious psychotic symptoms persisted. Finally the patient was treated by mECT in addition to anti-psychotics with a successful outcome. The patient is now continuously treated with anti-psychotics and maintenance mECT every month. Later, at 63 years old, the NMDAR antibody was detected in both the serum and the CSF of this patient. The hypocretin level in the CSF sample (92 pg/ml) was unchanged from the time of his diagnosis.

We found that 2 other narcolepsy patients (among 5 examined), who had severe psychotic symptoms occurring 3 to 30 years after the onset of narcolepsy, were positive for the antibody (Table, group B). These cases were hypocretin deficient, but no significant encephalitis signs except predominant psychotic symptoms, were noted. They were under stimulant medications, and their hallucinations and delusions were unchanged when the stimulants were withdrawn. Antipsychotics (3 out of 3 cases) and mECT (one case) were required to manage the psychotic symptoms.

We also tested anti-NMDAR antibody in 10 hypocretin deficient narcolepsy patients without psychotic symptoms, and found 2 antibody positive patients (15/f, 22/f). Although antibody positive cases were found both in narcolepsy with and without psychotic symptoms, an increase in antibody positivity in the patients with psychotic symptoms was suggested (p = 0.025, Chi-square test).

### (Group C) Psychiatry cases

In addition to these cases, we also found 4 antibody positive patients out of 51 patients with schizophrenia or schizo-affective disorders (group C). The neurological symptoms were mild in these cases, and mECT was effective in 3 cases. These 4 cases were female, two cases had convulsions (cases 7, 8), and two cases had ovarian tumors (cases 8, 10).

#### Case report

A 26-year-old female patient (case 10) had normal development during childhood [19]. She had no problems with friendships in elementary school and junior high school. She was aware of depressive symptoms when she was 16 years old, although she had no emotional stress. She was diagnosed with depression and received antidepressants from a clinic. Her diagnosis was changed to bipolar disorder because the patient presented a hypomanic episode at age 17. After that, her mood was stable and treatment was discontinued.

The patient had insomnia and hypobulia at age 22. The patient tried to jump out of a window (of the upper floor), and thus she was transferred to a closed ward. In this hospital, the patient exhibited a variety of symptoms including delusions of persecution and observation, self-injury (head banging) and substupor. There were no specific findings on brain MRI. The patient subsequently was treated with various anti-psychotics based on a schizophrenia diagnosis, however she was only in partial remission.

The patient had been treated with mECT (a total of ten times) since she was 23 years old, and her symptoms almost disappeared. Therefore her therapy focused on mECT, and she was transferred to our hospital. mECT was needed every other week continuously. The patient complained of atypical genital bleeding at age 24 years old, and an ovarian tumor was detected by abdominal ultrasonography. Pelvic MRI showed a cystic tumor in the right ovary with low T1 and high T2. The diameter and length were 4.7 × 3.4 cm and 4.3 cm, respectively.

She was positive for anti-NMDAR antibody. The patient underwent oophorectomy at age 26 years. The pathological diagnosis was ovarian cyst without teratoma. After the operation, she has been treated only with oral medication (antipsychotics) and follow-ups.

This patient showed atypical clinical history as a schizophrenic and resistance to pharmacological treatments, but responded relatively well to mECT. The percentage of NMDAR positive cases of this group C (4 out of 51 cases) is similar to that of Zandi’s report (3 out of 46 cases).

## Discussion

Our results showed a number of cases with NMDAR antibody positivity in a broader range of psychiatric disorders, such as sleep disorders and schizophrenia (group A: 3 out of 5 cases, B: 3 out of 5 cases & C: 4 out of 51 cases). Although the causative relationship between NMDAR antibody positivity and psychiatric symptoms in these patients are unknown, they exhibit unique demographic and clinical characteristics. Eight (out of 10) are female, the majority of cases are 20–30 year olds, and ovarian tumors are found in 2 patients. Most of their symptoms are resistant to pharmacological treatments but respond relatively well to mECT, the clinical characteristics often seen in psychotic symptoms associated with NMDAR encephalitis [22,23].

### NMDAR and psychiatric symptoms

Schizophrenia is a common, heterogeneous, and complex disorder with unknown aetiology [24]. There is established evidence of NMDAR hypofunction [25] as a central component of the functional dysconnectivity; this is one of the most accepted models for schizophrenia [26]. Moreover, autoimmune mechanisms have been proposed to be involved, at least in subgroups of schizophrenia patients [27,28]. In the last few years, a number of antibodies to neuronal cell surface antigens have been identified in cases of autoimmune encephalitis that respond to immunotherapy [29,30]. Over two-thirds of patients with NMDAR antibody encephalitis have prominent psychiatric symptoms or may present to psychiatric services in the first instance [23,29,31-33]. The psychiatric symptoms are those seen in schizophrenia including delusions, hallucinations, and catatonic movement disorder.

This characteristic clinical presentation resembles acute psychosis followed by a rapid decline in the level of consciousness, central hypoventilation, seizures, involuntary movements, and autonomic instability. Although anti-NMDAR encephalitis is a potentially fatal condition, if the diagnosis is made rapidly, effective treatments are available [29,34].

In fact, the most favorable outcome occurs with tumor removal (e.g., teratoma), usually in combination with immunotherapy (IV steroids, IV immunoglobulin, or plasma exchange) [9,35]. Also, a good clinical outcome was reported in patients treated with immunotherapy without tumor removal [36,37]. Besides tumor removal and immunotherapy, symptomatic treatment with antiepileptic drugs and benzodiazepines may partially relieve symptoms [5,36].

Our first three cases in group A had typical clinical pictures of anti-NMDAR encephalitis, beginning with psychiatric symptoms, followed by seizures and disturbances of consciousness (Table 1, group A). No tumors were found in these cases, but two of them responded well to steroid pulse treatments.

### Hypersomnia and encephalitis

As far as we know, there has been no report of narcolepsy with NMDAR antibody positivity. However, two studies reported that some patients with contemporary Encephalitis Lethergica (EL) were positive for NMDAR antibodies [23,38]. Ten out of twenty patients were positive and these patients predominantly fit into the dyskinetic form of EL [38]. The five patients with the somnolent-Parkinsonian form of EL, which is considered to be the classic form of EL, were negative for NMDAR antibodies [38].

These results together with the fact that 3 out of 5 narcoleptic subjects who were positive for anti-NMDAR antibodies exhibited severe psychiatric symptoms and that 8 out of 10 conventional narcoleptic subjects studied were negative for NMDAR antibodies (p = 0.025, Chi-square test), show that NMDAR antibody positivity may be more specifically related with occurrences of psychiatric symptoms.

Nevertheless, it is possible that the immune mediated mechanisms are more frequently involved in narcolepsy and that these may also be responsible for their associated symptoms, and further studies are warranted.

### The prevalence of NMDAR positivity in group C

The occurrence rate of NMDAR positive cases in our group C (4 out of 51 cases) is similar to that of Zandi’s report (3 out of 46 cases)[33].

Since there had been no reported cases of NMDAR antibodies identified in patients with purely psychiatric disorders, Zandi et al. [33] hypothesized that this antibody would be present in a proportion of patients with early schizophrenia, in the absence of overt seizures, movement disorders, or other neurological signs. They found 3 cases in 46 examined cases that fulfilled DSM IV criteria for schizophrenia, and the patients were tested early in the course of their illness. They also described the first case of a patient with NMDAR antibodies and a purely psychiatric presentation, that responded to immunotherapy (plasmapheresis, oral prednisolone).

### Atypical psychosis

The term, “atypical psychosis” has been used, especially by Japanese psychiatrists [39] as a possible clinical entity for acute and transient psychotic disorders which cannot be easily classified as either schizophrenia or a mood disorder with psychotic features. Some of the important clinical characteristics of atypical psychosis include acute onset, emotional disturbances, psychomotor disturbances, alternations of consciousness, high prevalence in women, and oriented premorbid personality, characteristics that mirror those of our psychotic cases. These authors had suspected involvements of brain organic changes in atypical psychosis.

However, atypical psychosis, by its meaning, comprises a widely varied and poorly understood collection of disorders, and atypical psychosis was listed in DSM-III-R under the heading Psychosis Not Otherwise Specified (NOS); this does not define the nosological entity and is rather used as a residual category. Consequently, in DSM-IV, the term atypical psychosis is no longer mentioned as a synonym for this category.

While schizophrenia and affective disorders have dominated the psychiatric literature and research efforts in psychotic disorders, several other atypical psychotic conditions are emerging as significant. Included among these are psychotic disorders secondary to medical conditions [40]. If NMDAR antibody positivity is functionally involved in pathophysiology of the disease in the patients listed in group C, the disease will fit in the category of psychotic disorders secondary to medical condition.

Together with our present study, further determination of whether the anti-NMDAR antibody plays functional roles in these patients with schizophrenia, schizo-affective disorders and atypical psychosis, is critical, since treatment choices, including immunotherapy, are different from those for classical psychosis and since the immune mediated mechanisms may also be involved more frequently in these psychotic patients.

### mECT effects

We present cases to illustrate that anti-NMDAR encephalitis should be considered when diagnosing patients with acute psychosis, and that mECT can possibly be considered as an effective treatment option for these cases.

In our 5 out of 10 anti-NMDAR antibody positive cases, mECT was effective. In one previous case series, one patient was found to respond to ECT [41]. Two patients have been reported with paraneoplastic catatonia and ovarian teratoma that partially improved with ECT, but full recovery was only obtained after tumor removal [9,22]. Still, the remission may have been spontaneous and the temporal association between ECT and recovery may have been coincidental. Recently, Braakman [23] reported that one patient (47 old male) deteriorated clinically in a period of 10 weeks, and recovered in a period of 3 weeks after ECT was started.

The mechanism of action of ECT remains largely unclear. Still, in animal models ECT has been shown to up-regulate NMDA receptors [42]. This may, in part, explain the efficacy of ECT in our patients, since NMDAR was down-regulated during periods of anti-NMDAR encephalitis [34].

We report remarkable recoveries of our patients following mECT. Psychosis, delusions, stupor, and catatonia rapidly disappeared. Further clinical observation or studies in patients with anti-NMDAR encephalitis are needed to determine the significance of our observations [38].

Results of our study together with those by others redefine this new class of psychotic disorders positive for anti- NMDAR antibody. These include narcolepsy with psychosis, EL [38], schizophrenia accompanied by convulsion [13], atypical symptoms of psychosis and psychotic patients who are drug resistant but respond relatively well to mECT.

We are also aware of the limitations of the study. (1) We did not include normal controls or typical schizophrenia subjects. (2) The retrospective recollection of the clinical data may favor the description of partial or isolated symptoms of the disease because we could miss subtle signs or symptoms associated with the predominant manifestation. (3) We did not measure the CSF antibody in the majority of our cases and future prospective studies should include paired serum-CSF antibody measures.

## Conclusions

In addition to 3 typical cases with NMDAR encephalitis, we found 7 cases with anti-NMDAR antibody associated with various psychotic and sleep symptoms, which lack any noticeable clinical signs of encephalitis (seizures and autonomic symptoms) throughout the course of the disease episodes. These patients exhibited two distinct clinical characteristics; narcolepsy with severe psychosis (3 cases) or schizophrenia (4 cases). Further determination if the anti-NMDAR antibody plays functional roles in these patients is essential, since the immune mediated mechanisms may be involved more frequently in nonencephalitic atypical psychosis, schizophrenia accompanied with convulsion and sleep disorders than currently thought.

## Competing interests

The authors declare that they have no competing interests.

## Authors' contributions

Concept: KT, TK, TS; Data collection: KT, SB, WI, JK, AM; Data analysis: TK, KT, SN; First draft: KT, TK, YH, TS, SN; Final revision: KT, TK, YH, TS, SN; All authors read and approved the final manuscript.

## Pre-publication history

The pre-publication history for this paper can be accessed here:

http://www.biomedcentral.com/1471-244X/12/37/prepub
